# 2-(4-Bromo­phen­yl)-3,4-dihydro­isoquinolin-2-ium thio­cyanate hemihydrate

**DOI:** 10.1107/S1600536811038542

**Published:** 2011-09-30

**Authors:** Yanni Ma, Fangjun Cao, Bin Zhu, Weigang Hu, Le Zhou

**Affiliations:** a College of Science, Northwest Agriculture and Forest University, Yangling 712100, People’s Republic of China

## Abstract

In the title hemihydrated salt, C_15_H_13_BrN^+^·NCS^−^·0.5H_2_O, the two benzene rings are aligned at a dihedral angle of 46.9 (1)°. The six-membered heterocycle of the dihydro­isoquinoline unit adopts a half-chair conformation. The water mol­ecule and thio­cyanate ion are linked by O—H⋯N hydrogen bonds, generating a four-membered ring motif. In addition, C—H⋯O and C—H⋯S inter­actions link the components into a chain along the *c* axis. π–π inter­actions [centroid–centroid distance = 3.974 (2) Å] link the chains into sheets and further π—π [centroid–centroid distance = 3.746 (2) Å] and C—H⋯π inter­actions give rise to a three-dimensional nework.

## Related literature

For the synthesis of the title compound, see: Ishii *et al.* (1985 ▶). For the biological activity of tetra­hydro­isoquinoline derivatives, see: Abe *et al.* (2005 ▶); Kamal *et al.* (2011 ▶); Lane *et al.* (2006 ▶); Liu *et al.* (2009) ▶; Storch *et al.* (2002) ▶; Jang *et al.* (2009) ▶. 
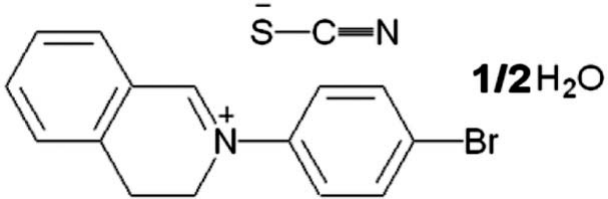


## Experimental

### 

#### Crystal data

C_15_H_13_BrN^+^·NCS^−^·0.5H_2_O
*M* *_r_* = 354.26
Triclinic, 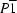

*a* = 9.0211 (12) Å
*b* = 9.2685 (12) Å
*c* = 10.7284 (14) Å
α = 81.174 (2)°
β = 66.699 (1)°
γ = 68.368 (1)°
*V* = 765.81 (17) Å^3^
*Z* = 2
Mo *K*α radiation
μ = 2.82 mm^−1^
*T* = 296 K
0.50 × 0.41 × 0.37 mm

#### Data collection

Bruker SMART APEXII CCD area-detector diffractometer
Absorption correction: multi-scan (*SADABS*; Sheldrick, 1996 ▶) *T*_min_ = 0.333, *T*_max_ = 0.422
5705 measured reflections
2824 independent reflections
2262 reflections with *I* > 2σ(*I*)
*R*_int_ = 0.015

#### Refinement

*R*[*F* ^2^ > 2σ(*F* ^2^)] = 0.034
*wR*(*F* ^2^) = 0.100
*S* = 1.04
2824 reflections
190 parameters
H-atom parameters constrained
Δρ_max_ = 0.54 e Å^−3^
Δρ_min_ = −0.53 e Å^−3^

### 

Data collection: *APEX2* (Bruker, 2004 ▶); cell refinement: *SAINT* (Bruker, 2004 ▶); data reduction: *SAINT*; program(s) used to solve structure: *SHELXS97* (Sheldrick, 2008 ▶); program(s) used to refine structure: *SHELXL97* (Sheldrick, 2008 ▶); molecular graphics: *SHELXTL* (Sheldrick, 2008 ▶); software used to prepare material for publication: *SHELXTL*.

## Supplementary Material

Crystal structure: contains datablock(s) global, I. DOI: 10.1107/S1600536811038542/ng5230sup1.cif

Structure factors: contains datablock(s) I. DOI: 10.1107/S1600536811038542/ng5230Isup2.hkl

Supplementary material file. DOI: 10.1107/S1600536811038542/ng5230Isup3.cml

Additional supplementary materials:  crystallographic information; 3D view; checkCIF report

## Figures and Tables

**Table 1:** Hydrogen-bond geometry (Å, °) *Cg*2 is the centroid of the C1–C6 ring.

*D*—H⋯*A*	*D*—H	H⋯*A*	*D*⋯*A*	*D*—H⋯*A*
O1—H1*W*⋯N2	0.85	1.83	2.642 (8)	159
O1—H2*W*⋯N2^i^	0.85	2.04	2.879 (9)	171
C5—H5⋯O1^ii^	0.93	2.60	3.133 (8)	117
C9—H9⋯S1	0.93	2.81	3.709 (3)	162
C12—H12⋯O1^i^	0.93	2.57	3.438 (8)	156
C14—H14⋯*Cg*2^iii^	0.93	2.87	3.447 (4)	121

Symmetry codes: (i) 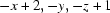; (ii) 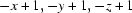; (iii) 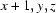.

**Fig. 1.**
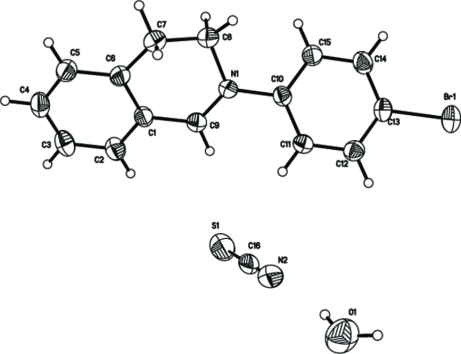
An ORTEP drawing (30% probability displacement ellipsoids) of a single molecule of the title compound.

**Fig. 2.**
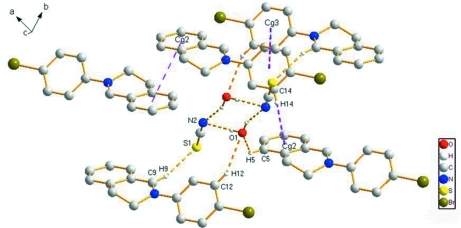
The three-dimension structure of the title compound.

## supplementary crystallographic information

### Comment

Tetrahydroisoquinoline derivatives have recently attracted a lot of interest according to their outstanding bioactivity (Abe *et al.*, 2005; Storch *et al.*, 2002, Jang *et al.*, 2009; Lane *et al.*, 2006; Kamal *et al.*, 2011; Liu *et al.*, 2009). Considering the importance of these compounds, we prepared some tetrahydroisoquinoline derivatives. The title compound is an unexpected salt.

In the title hemihydrated salt, C_16_H_14_BrN_2_O_0.5_S, the two benzene rings are aligned at 46.9 (1)°. The six-membered heterocycle of the dihydroisoquinoline unit adopts a half-chair conformation. The lattice water and thiocyanate ion are linked by O—H···N hydrogen bonds to generateing four-membered ring motifs. Additionally, C—H···O and C—H···S interactions link the ions into a chain along *c* axis; π—π interactions link the chains into sheets, and other π—π and C—H···π interactions give rise to a three-dimension nework structure. The *Cg*2···*Cg*2 (1 - *x*, 2 - *y*, -*z*) distance is 3.974 (2) Å. The *Cg*3···*Cg*3 (2 - *x*, 1 - *y*, 1 - *z*) distance is 3.7457 (18) Å.

### Experimental

The title compound was synthesized according to the literature procedure (Ishii *et al.*, 1985), and the single crystals were obtained from its solution of dichloromethane-petroleum ether by slow evaporation at room temperature.

### Refinement

The positions and isotropic displacement parameters of the water H atoms, H1W and H2W, were placed geometrically. The other H atoms were positioned geometrically and allowed to ride on their parent atoms at distances of 0.93(aromatic CH) or 0.97 Å (methylene CH_2_), with *U*_iso_(H) = 1.2*U*_eq_(C). The water molecule is of 0.5 occupany as it is close to a center of inversion.

### Crystal data

**Table:**

C_15_H_13_BrN^+^·CNS^−^·0.5H_2_O	*Z* = 2
*M**_r_* = 354.26	*F*(000) = 358
Triclinic, *P*1	*D*_x_ = 1.536 Mg m^−^^3^
*a* = 9.0211 (12) Å	Mo *K*α radiation, λ = 0.71073 Å
*b* = 9.2685 (12) Å	Cell parameters from 2388 reflections
*c* = 10.7284 (14) Å	θ = 2.6–25.5°
α = 81.174 (2)°	µ = 2.82 mm^−^^1^
β = 66.699 (1)°	*T* = 296 K
γ = 68.368 (1)°	Block, yellow
*V* = 765.81 (17) Å^3^	0.50 × 0.41 × 0.37 mm

### Data collection

**Table:**

Bruker SMART APEXII CCD area-detector diffractometer	2824 independent reflections
Radiation source: fine-focus sealed tube	2262 reflections with *I* > 2σ(*I*)
graphite	*R*_int_ = 0.015
phi and ω scans	θ_max_ = 25.5°, θ_min_ = 2.6°
Absorption correction: multi-scan (*SADABS*; Sheldrick, 1996)	*h* = −10→10
*T*_min_ = 0.333, *T*_max_ = 0.422	*k* = −11→11
5705 measured reflections	*l* = −12→12

### Refinement

**Table:**

Refinement on *F*^2^	Primary atom site location: structure-invariant direct methods
Least-squares matrix: full	Secondary atom site location: difference Fourier map
*R*[*F*^2^ > 2σ(*F*^2^)] = 0.034	Hydrogen site location: inferred from neighbouring sites
*wR*(*F*^2^) = 0.100	H-atom parameters constrained
*S* = 1.04	*w* = 1/[σ^2^(*F*_o_^2^) + (0.0542*P*)^2^ + 0.2978*P*] where *P* = (*F*_o_^2^ + 2*F*_c_^2^)/3
2824 reflections	(Δ/σ)_max_ < 0.001
190 parameters	Δρ_max_ = 0.54 e Å^−^^3^
0 restraints	Δρ_min_ = −0.53 e Å^−^^3^

### Special details

**Table:**

Geometry. All e.s.d.'s (except the e.s.d. in the dihedral angle between two l.s. planes)are estimated using the full covariance matrix. The cell e.s.d.'s are takeninto account individually in the estimation of e.s.d.'s in distances, anglesand torsion angles; correlations between e.s.d.'s in cell parameters are onlyused when they are defined by crystal symmetry. An approximate (isotropic)treatment of cell e.s.d.'s is used for estimating e.s.d.'s involving l.s. planes.
Refinement. Refinement of *F*^2^ against ALL reflections. The weighted *R*-factor *wR* and goodness of fit *S* are based on *F*^2^, conventional *R*-factors *R* are based on *F*, with *F* set to zero for negative *F*^2^. The threshold expression of *F*^2^ > σ(*F*^2^) is used only for calculating *R*-factors(gt) *etc*. and is not relevant to the choice of reflections for refinement. *R*-factors based on *F*^2^ are statistically about twice as large as those based on *F*, and *R*- factors based on ALL data will be even larger.

### Fractional atomic coordinates and isotropic or equivalent isotropic displacement parameters (Å^2^)

**Table:**

	*x*	*y*	*z*	*U*_iso_*/*U*_eq_	Occ. (<1)
S1	0.93320 (15)	0.24168 (14)	0.06970 (15)	0.0945 (4)	
N2	0.8517 (6)	0.1537 (4)	0.3422 (5)	0.0945 (12)	
C16	0.8864 (5)	0.1895 (4)	0.2255 (6)	0.0872 (15)	
O1	0.8821 (10)	−0.0376 (8)	0.5473 (7)	0.116 (2)	0.50
H1W	0.9000	0.0172	0.4752	0.174*	0.50
H2W	0.9526	−0.0671	0.5878	0.174*	0.50
C1	0.6164 (4)	0.7282 (3)	0.1329 (3)	0.0490 (7)	
C2	0.5976 (4)	0.6630 (4)	0.0343 (3)	0.0606 (8)	
H2	0.6783	0.5695	−0.0052	0.073*	
C3	0.4598 (5)	0.7367 (5)	−0.0051 (3)	0.0662 (9)	
H3	0.4470	0.6930	−0.0711	0.079*	
C4	0.3401 (4)	0.8761 (4)	0.0538 (4)	0.0632 (9)	
H4	0.2474	0.9260	0.0267	0.076*	
C5	0.3568 (4)	0.9415 (4)	0.1519 (4)	0.0598 (8)	
H5	0.2751	1.0350	0.1908	0.072*	
C6	0.4943 (4)	0.8696 (3)	0.1936 (3)	0.0515 (7)	
C7	0.5176 (4)	0.9260 (4)	0.3058 (4)	0.0660 (9)	
H7A	0.4658	1.0382	0.3104	0.079*	
H7B	0.4580	0.8836	0.3913	0.079*	
C8	0.7013 (5)	0.8819 (4)	0.2877 (4)	0.0629 (9)	
H8A	0.7538	0.9462	0.2169	0.076*	
H8B	0.7081	0.9013	0.3713	0.076*	
C9	0.7584 (4)	0.6529 (3)	0.1743 (3)	0.0491 (7)	
H9	0.8271	0.5522	0.1445	0.059*	
C10	0.9469 (4)	0.6359 (3)	0.2869 (3)	0.0449 (6)	
C11	0.9916 (4)	0.4784 (3)	0.3128 (3)	0.0494 (7)	
H11	0.9240	0.4245	0.3102	0.059*	
C12	1.1367 (4)	0.4012 (3)	0.3427 (3)	0.0527 (7)	
H12	1.1687	0.2947	0.3591	0.063*	
C13	1.2341 (4)	0.4834 (4)	0.3479 (3)	0.0507 (7)	
C14	1.1875 (4)	0.6411 (4)	0.3261 (3)	0.0606 (8)	
H14	1.2528	0.6953	0.3324	0.073*	
C15	1.0429 (4)	0.7188 (4)	0.2946 (3)	0.0581 (8)	
H15	1.0108	0.8254	0.2787	0.070*	
N1	0.7980 (3)	0.7159 (3)	0.2511 (2)	0.0453 (5)	
Br1	1.43677 (4)	0.37833 (4)	0.38559 (4)	0.06978 (17)	

### Atomic displacement parameters (Å^2^)

**Table:**

	*U*^11^	*U*^22^	*U*^33^	*U*^12^	*U*^13^	*U*^23^
S1	0.0726 (7)	0.0938 (8)	0.1153 (9)	−0.0129 (6)	−0.0337 (6)	−0.0381 (7)
N2	0.128 (3)	0.070 (2)	0.126 (3)	−0.049 (2)	−0.083 (3)	0.028 (2)
C16	0.077 (3)	0.0439 (19)	0.169 (5)	−0.0073 (17)	−0.079 (3)	−0.017 (3)
O1	0.129 (6)	0.122 (5)	0.094 (4)	−0.048 (5)	−0.043 (4)	0.025 (4)
C1	0.0478 (16)	0.0527 (16)	0.0527 (17)	−0.0200 (13)	−0.0231 (13)	0.0025 (13)
C2	0.0553 (18)	0.071 (2)	0.0596 (19)	−0.0198 (16)	−0.0237 (16)	−0.0079 (16)
C3	0.063 (2)	0.093 (3)	0.0567 (19)	−0.035 (2)	−0.0304 (17)	0.0040 (18)
C4	0.0551 (19)	0.075 (2)	0.070 (2)	−0.0301 (18)	−0.0341 (17)	0.0235 (18)
C5	0.0534 (18)	0.0529 (17)	0.075 (2)	−0.0188 (14)	−0.0284 (16)	0.0091 (16)
C6	0.0513 (17)	0.0452 (16)	0.0610 (18)	−0.0181 (13)	−0.0249 (14)	0.0068 (13)
C7	0.063 (2)	0.0480 (17)	0.089 (3)	−0.0078 (15)	−0.0378 (19)	−0.0109 (17)
C8	0.072 (2)	0.0457 (17)	0.081 (2)	−0.0122 (15)	−0.0429 (19)	−0.0071 (15)
C9	0.0499 (16)	0.0464 (16)	0.0522 (17)	−0.0148 (13)	−0.0202 (14)	−0.0041 (13)
C10	0.0473 (15)	0.0500 (16)	0.0427 (15)	−0.0196 (13)	−0.0201 (12)	0.0019 (12)
C11	0.0534 (16)	0.0449 (16)	0.0541 (17)	−0.0156 (13)	−0.0229 (14)	−0.0059 (13)
C12	0.0562 (18)	0.0475 (16)	0.0515 (17)	−0.0120 (14)	−0.0203 (14)	−0.0055 (13)
C13	0.0446 (15)	0.0642 (19)	0.0420 (15)	−0.0158 (14)	−0.0181 (13)	0.0023 (13)
C14	0.064 (2)	0.069 (2)	0.069 (2)	−0.0383 (17)	−0.0360 (17)	0.0174 (16)
C15	0.066 (2)	0.0523 (17)	0.071 (2)	−0.0303 (15)	−0.0380 (17)	0.0173 (15)
N1	0.0501 (13)	0.0415 (12)	0.0505 (13)	−0.0173 (10)	−0.0235 (11)	0.0005 (10)
Br1	0.0550 (2)	0.0851 (3)	0.0708 (3)	−0.01661 (17)	−0.03359 (17)	0.00511 (18)

### Geometric parameters (Å, °)

**Table:**

S1—C16	1.597 (6)	C7—H7B	0.9700
N2—C16	1.189 (6)	C8—N1	1.486 (4)
N2—O1	2.642 (8)	C8—H8A	0.9700
O1—H1W	0.8500	C8—H8B	0.9700
O1—H2W	0.8500	C9—N1	1.297 (4)
C1—C2	1.388 (4)	C9—H9	0.9300
C1—C6	1.409 (4)	C10—C11	1.379 (4)
C1—C9	1.422 (4)	C10—C15	1.384 (4)
C2—C3	1.376 (5)	C10—N1	1.444 (3)
C2—H2	0.9300	C11—C12	1.379 (4)
C3—C4	1.385 (5)	C11—H11	0.9300
C3—H3	0.9300	C12—C13	1.379 (4)
C4—C5	1.374 (5)	C12—H12	0.9300
C4—H4	0.9300	C13—C14	1.373 (4)
C5—C6	1.386 (4)	C13—Br1	1.899 (3)
C5—H5	0.9300	C14—C15	1.384 (4)
C6—C7	1.497 (5)	C14—H14	0.9300
C7—C8	1.490 (5)	C15—H15	0.9300
C7—H7A	0.9700		
			
C16—N2—O1	154.6 (4)	N1—C8—H8A	109.2
N2—C16—S1	178.6 (4)	C7—C8—H8A	109.2
N2—O1—H1W	14.7	N1—C8—H8B	109.2
N2—O1—H2W	134.1	C7—C8—H8B	109.2
H1W—O1—H2W	120.7	H8A—C8—H8B	107.9
C2—C1—C6	120.3 (3)	N1—C9—C1	124.0 (3)
C2—C1—C9	120.5 (3)	N1—C9—H9	118.0
C6—C1—C9	119.2 (3)	C1—C9—H9	118.0
C3—C2—C1	120.1 (3)	C11—C10—C15	120.9 (3)
C3—C2—H2	120.0	C11—C10—N1	119.8 (2)
C1—C2—H2	120.0	C15—C10—N1	119.4 (2)
C2—C3—C4	119.7 (3)	C12—C11—C10	119.8 (3)
C2—C3—H3	120.1	C12—C11—H11	120.1
C4—C3—H3	120.1	C10—C11—H11	120.1
C5—C4—C3	120.8 (3)	C11—C12—C13	119.3 (3)
C5—C4—H4	119.6	C11—C12—H12	120.4
C3—C4—H4	119.6	C13—C12—H12	120.4
C4—C5—C6	120.6 (3)	C14—C13—C12	121.2 (3)
C4—C5—H5	119.7	C14—C13—Br1	118.9 (2)
C6—C5—H5	119.7	C12—C13—Br1	119.9 (2)
C5—C6—C1	118.5 (3)	C13—C14—C15	119.7 (3)
C5—C6—C7	124.6 (3)	C13—C14—H14	120.2
C1—C6—C7	116.8 (3)	C15—C14—H14	120.2
C8—C7—C6	113.0 (3)	C10—C15—C14	119.2 (3)
C8—C7—H7A	109.0	C10—C15—H15	120.4
C6—C7—H7A	109.0	C14—C15—H15	120.4
C8—C7—H7B	109.0	C9—N1—C10	121.8 (2)
C6—C7—H7B	109.0	C9—N1—C8	118.7 (2)
H7A—C7—H7B	107.8	C10—N1—C8	118.9 (2)
N1—C8—C7	111.9 (3)		
			
O1—N2—C16—S1	−134 (17)	N1—C10—C11—C12	177.9 (3)
C6—C1—C2—C3	0.3 (5)	C10—C11—C12—C13	0.8 (4)
C9—C1—C2—C3	179.9 (3)	C11—C12—C13—C14	1.0 (5)
C1—C2—C3—C4	0.1 (5)	C11—C12—C13—Br1	−178.8 (2)
C2—C3—C4—C5	−0.4 (5)	C12—C13—C14—C15	−1.8 (5)
C3—C4—C5—C6	0.3 (5)	Br1—C13—C14—C15	178.1 (3)
C4—C5—C6—C1	0.1 (5)	C11—C10—C15—C14	1.3 (5)
C4—C5—C6—C7	−175.8 (3)	N1—C10—C15—C14	−178.6 (3)
C2—C1—C6—C5	−0.4 (4)	C13—C14—C15—C10	0.6 (5)
C9—C1—C6—C5	179.9 (3)	C1—C9—N1—C10	−178.1 (3)
C2—C1—C6—C7	175.8 (3)	C1—C9—N1—C8	−6.4 (4)
C9—C1—C6—C7	−3.8 (4)	C11—C10—N1—C9	−37.9 (4)
C5—C6—C7—C8	−151.8 (3)	C15—C10—N1—C9	142.1 (3)
C1—C6—C7—C8	32.3 (4)	C11—C10—N1—C8	150.5 (3)
C6—C7—C8—N1	−46.7 (4)	C15—C10—N1—C8	−29.6 (4)
C2—C1—C9—N1	170.0 (3)	C7—C8—N1—C9	35.1 (4)
C6—C1—C9—N1	−10.3 (4)	C7—C8—N1—C10	−152.9 (3)
C15—C10—C11—C12	−2.0 (4)		

### Hydrogen-bond geometry (Å, °)

**Table:**

Cg2 is the centroid of the C1–C6 ring.

**Table:**

*D*—H···*A*	*D*—H	H···*A*	*D*···*A*	*D*—H···*A*
O1—H1W···N2	0.85	1.83	2.642 (8)	159.
O1—H2W···N2^i^	0.85	2.04	2.879 (9)	171.
C5—H5···O1^ii^	0.93	2.60	3.133 (8)	117
C9—H9···S1	0.93	2.81	3.709 (3)	162
C12—H12···O1^i^	0.93	2.57	3.438 (8)	156
C14—H14···Cg2^iii^	0.93	2.87	3.447 (4)	121

Symmetry codes: (i) −*x*+2, −*y*, −*z*+1; (ii) −*x*+1, −*y*+1, −*z*+1; (iii) *x*+1, *y*, *z*.
